# Addressing Cognitive Function and Psychological Well-Being in Chronic Kidney Disease: A Systematic Review on the Use of Technology-Based Interventions

**DOI:** 10.3390/ijerph20043342

**Published:** 2023-02-14

**Authors:** Alexandra-Elena Marin, Rosa Redolat, José-Antonio Gil-Gómez, Patricia Mesa-Gresa

**Affiliations:** 1 Department of Psychobiology, Faculty of Psychology and Logopedics, Universitat de València, 46010 Valencia, Spain; 2 Instituto Universitario de Automática e Informática Industrial, Universitat Politècnica de València, 46022 Valencia, Spain

**Keywords:** chronic kidney disease, technology-based intervention, cognition, psychological well-being, systematic review

## Abstract

Patients with chronic kidney disease (CKD) are at risk of both a gradual decline in cognitive function and an increase in psychological distress. This includes symptoms of anxiety, depression, and sleep disturbances, all of which are factors that have been associated with increased morbidity and mortality. In response, we are now seeing that interventions based on new digital technologies are increasingly used in order to optimize patients’ quality of life. Systematic research of the literature on electronic databases (MEDLINE/PubMed, Scopus, Web of Science, and PsycInfo/ProQuest) covering the period from 2012 to 2022 was conducted in order to methodically review the existing evidence regarding the implementation and effectiveness of technology-based interventions in the management of cognitive and psychological well-being symptoms in patients with CKD. A total of 739 articles were retrieved, 13 of which are included in the present review. All the studies focused on the usability, acceptability, and feasibility of technology-based interventions aimed at psychological symptoms, with no studies targeting cognitive functioning. Technology-based interventions offer feelings of safety, fun, and satisfaction, and they also have the potential to improve CKD patients’ health outcomes regarding their psychological well-being. The diverseness of technologies allows an approximation towards the identification of those types of technologies most frequently used, as well as the symptoms targeted. There was considerable heterogeneity in the types of technologies used for interventions in so few studies, making it difficult to draw conclusive findings with regard to their efficiency. In order to adequately assess the technology-based health interventions effect, future lines of research should consider designing non-pharmacological treatments for the improvement of cognitive and psychological symptoms in this type of patient.

## 1. Introduction

### Background

Chronic kidney disease (CKD) is a growing health problem worldwide, with over 800 million people affected [1]. According to Kalantar-Zadeh et al. [2], CKD can be identified according to the degree of kidney damage suffered by the patient or by a measured glomerular filtration rate of ≤60 mL/min/1.73 m^2^ for more than 3 months. Depending on the decline of this rate, the disease can be classified into five stages, with most people falling into the category of stages 3–5 [3]. The progression of CKD throughout the stages is evident, with early-stage CKD being generally asymptomatic, to stage five, also called end-stage renal disease (ESRD), where patients need kidney replacement therapy in order to sustain life [4]. There are three types of kidney replacement therapy—peritoneal dialysis, hemodialysis (HD), and kidney transplant—with approximately 2.62 million people worldwide undergoing HD treatment [5]. While acknowledging that kidney replacement therapy is indispensable for life-preserving, it also involves multidisciplinary teamwork composed of clinicians with different specializations, researchers, and engineers. This implies a large economic investment in the health systems, which can represent a huge burden for many countries, especially those with low- to middle-income.

Increased severity of CKD is associated with a gradual decline in cognitive function, especially in processing, memory, and executive function, including cognitive domains related to task planning and performance [6,7]. This implies that patients in the final stages of their kidney disease are likely to experience deficits in memory, concentration, and planning that could ultimately impair their ability to participate in their health care, thus affecting medication adherence, diet modifications, quality of life, and the ability to comprehend and provide consent for medical procedures [6,8]. The negative impact of cognitive decline on quality of life and emotional well-being is significant, directly correlating with frailty, depression, and an increased risk of days spent in hospital and mortality, all factors which contribute to the individual, the social and economic burden of CKD mentioned previously [9].

In the last years, more attention has been paid to non-renal symptoms of CKD, discovering that certain factors such as the changing role within the family, the decrease in physical activity, and the medication treatment can certainly contribute to the development of depressive symptoms [10,11]. Furthermore, it has been hypothesized that depression and ESRD interact at three different levels: first, depression may contribute to the progression of CKD into ESRD through parallel inflammatory pathways; second, the decrease in quality of life and poor well-being associated with ESRD can lead to depression; third, depression is related to poorer disease outcomes due to poor nutrition and lack of adherence to treatment [12,13,14].

With regard to anxiety disorders, there is less literature on their prevalence in CKD patients, although it is also common for anxiety symptoms to coexist with depressive symptoms in patients, mainly in the more advanced phases of the disease [15]. The prevalence rate of anxiety in CKD patients is approximately 38%, with the most common disorder being generalized anxiety disorder, affecting 30–45% of patients [16].

Sleep disorders are very common in patients with CKD, especially in the most advanced stages of the disease when dialysis treatment is being received. It has been estimated that 50–80% of hemodialysis patients show some type of sleep disturbance, including restless legs syndrome, insomnia, apnea, frequent awakenings during the day, or daytime sleepiness, among other problems that directly affect physical health and quality of life of these patients, in addition to their emotional state and cognitive functioning [9,17,18]. The appearance of this type of disorder may be related to factors of the disease itself and its treatment, psychological factors, and factors related to the patient’s lifestyle [17].

Within ESRD, it has been seen that every kidney replacement therapy method has some requirements, benefits, and considerations. For example, while it is true that these procedures may help patients, they can also be considered a source of stress. HD treatment is not flexible, and patients must already deal with various aspects of the disease, even though they often find it difficult to cope with the prior stressors involved. Some of the most common stressors include physical dependence on the devices, limitations in physical and sexual functions, having to take a great number of medications, and loss of appetite and energy [10]. Likewise, patients are frequently exposed to the psychosocial stressors of job loss, loss of independence, changes in self-perception and self-concept, or fear of death. Therefore, restricting the daily activities that patients can perform could negatively affect their independence, financial aspects, and changes in role and self-esteem [10,19].

When designing treatment methods aimed at mitigating stress, anxiety, and depression in HD patients, it has been reported that pharmacological treatment alone does not appear to be a viable option for them, as patients are reluctant to accept it for managing symptoms. As well as this, pharmacotherapy may even impair quality of life due to side effects or medication interactions [20,21]. Instead, there are several techniques that are considered to be useful, such as cognitive-behavioral therapy [22,23,24], regular exercise [16,25], and breathing or relaxation techniques [26,27]. In addition, performing intradialytic exercise training programs has a positive effect on the physical and psychological functioning of patients [28,29,30].

New digital technology can help address cognitive and psychological symptoms through the design of interventions that are viable for symptom management while also being accessible, safe, and interactive [31,32]. For instance, virtual reality (VR), a computer-simulated real or imagined 3D environment, which allows users to experience the sensation of being present in a different physical space [33,34] has been used in different medical settings, such as people diagnosed with dementia and mild cognitive impairment [35,36,37,38]. Interventions delivered through VR have proved to be useful for patients with neurocognitive disorders by improving cognitive domains (e.g., memory, dual tasking, and visual attention) and psychological functioning (reduction of anxiety, increased use of coping strategies, and higher levels of well-being) [39]. VR is also used as a tool to play action video games, also called exergames. These serious games involve physical and cognitive demands in the manner of dual tasks, considered non-immersive VR games [40].

Electronic health (eHealth) technology can also help patients with their health-related objectives and has been used in other diseases in order to promote smoking cessation [41], reduce depressive symptoms in the general population [42], reduce fatigue in cancer survivors [43] and increase self-management support in coronary heart disease [44]. In addition, several studies reported the use of eHealth interventions for CKD, addressing depressive symptoms [22,45] and nutritional aspects [46,47].

New technologies have been incorporated into the treatment of various diseases, including CKD, and this trend has been reinforced in the wake of the COVID-19 pandemic [48]. However, to our knowledge, no previous systematic reviews have been conducted on the effects of technology-based interventions on cognitive and psychological well-being symptoms in patients with CKD. In view of these issues and based on the relevance of the psychological and cognitive symptomatology that accompanies patients with CKD, the main objective of the present article is to carry out a systematic review of the available evidence on technology-based interventions for CKD patients, addressing cognitive and psychological symptoms. Specifically, we aimed to review the following: (1) study characteristics and type of technology used; (2) intervention implemented related to psychological well-being or cognitive symptoms and their possible contribution to the results found; (3) the effect outcomes; and (4) determinants of implementation.

## 2. Methods

### 2.1. Search Protocol

This review was performed according to the Preferred Reporting Items for Systematic Reviews and Meta-Analyses (PRISMA) statement [49], and it includes the updates described in PRISMA Declaration 2020 [50] for the search diagram.

### 2.2. Search Strategy and Information Sources

In September 2022, a systematic search was conducted to identify relevant articles in 4 electronic databases specialized in health sciences: MEDLINE/PubMed, Scopus, Web of Science, and PsycInfo/ProQuest [51]. In all of them, similar filters were applied depending on the available options, and search terms covered 3 different areas of interest: (1) CKD; (2) new technologies; and (3) psychological well-being and/or cognitive symptoms. Conclusively, the string of keywords used was (“kidney disease” or “chronic kidney disease” or “renal failure” or “renal disease” or “kidney failure” or “dialysis” or “hemodialysis” or “peritoneal dialysis” or “renal insufficiency” or “chronic renal disease” or “chronic kidney failure” or “kidney injury” or “kidney disorder” or “renal injury” or “renal disorder” or “renal dialysis”) and (“virtual reality” or “apps” or “mHealth” or “eHealth” or “mobile devices” or “wearable” or “digital health” or “video game” or “computer game” or “digital game” or “electronic game” or “assistive technology” or “artificial intelligence” or “voice assistant” or “augmented reality” or “telemedicine”) and (“intervention” or “skills” or “cognition” or “cognitive impairment” or “mental health” or “cognitive decline” or “psychotherapy” or “psychological wellbeing” or “wellbeing” or “empowerment” or “executive functions” or “memory” or “attention” or “anxiety” or “depression” or “mindfulness” or “psychotherapies” or “behavior therapy” or “cognitive behavioral therapy” or “color therapy” or “music therapy” or “psychosocial intervention” or “art therapy” or “awareness” or “consciousness” or “neurocognitive disorders” or “cognitive dysfunction”). Reference lists of the included studies were also searched to identify other relevant articles, and reference manager software was used throughout the studies—Mendeley Reference Manager.

### 2.3. Screening and Eligibility Criteria

Eligibility of the studies followed inclusion criteria: (1) interventions using eHealth technologies in adults with CKD, including patients undergoing hemodialysis treatment; (2) studies assessing effects of the intervention on patients’ psychological (e.g., quality of life, depression, anxiety, self-awareness, self-care) or cognitive symptoms; (3) studies available in the English language; (4) articles published between 2012 and 2022 (taking into account the fact that the objective of this review has to do with technological progress. Due to the recent developments in the fields of new technologies, it was considered appropriate to include articles starting in 2012 since this area of research has experienced an exponential increase in the last few years). Studies based on the following characteristics were excluded from this review: (1) studies focusing on family members, carers, or health care professionals of individuals with kidney failure or outcome measures that focused on family members, carers, or health care professionals of individuals with kidney disease; (2) research protocols and reviews; and (3) conference papers and abstracts.

### 2.4. Study Identification and Selection

Titles and abstracts were screened by two independent reviewers based on the eligibility criteria. Studies meeting the inclusion criteria, or studies that were unclear, were retained for full-text review. Discrepancies were resolved by consensus and a third reviewer for determination.

### 2.5. Data Extraction and Synthesis

A reviewer extracted the following information from each study: publication data (i.e., author, year, and title); study design; objectives; setting; participants (e.g., sample size, mean age, sex, diagnosis, and the demographic information for control groups when available); type of eHealth technology used for intervention (i.e., name of technology used, the number of sessions, as well as frequency and length of each session); outcome measures; results; and the general conclusion. A critical analysis of the literature was performed based on a descriptive numerical summary based on the characteristics of the studies, samples, symptoms assessed, and type of technologies.

### 2.6. Methodological Quality Assessment

Two independent reviewers (A.-E.M. and P.M.-G.) assessed the methodological quality of the included studies. Two additional reviewers (R.R. and J.-A.G.-G.) were consulted when necessary. Whenever it was needed, discrepancies were resolved through discussion and consensus.

In order to assess the risk of bias in all the studies included in this systematic review, The Critical Appraisal Checklist for Randomized Controlled Trials (RCTs) was used, as instructed by Moola et al. [52]. This checklist consists of 13 questions that assess different aspects of the study, such as compliance with the follow-up, the validity of the randomization, or the appropriate use of statistical analysis.

## 3. Results

### 3.1. Study Selection

**Figure 1:**
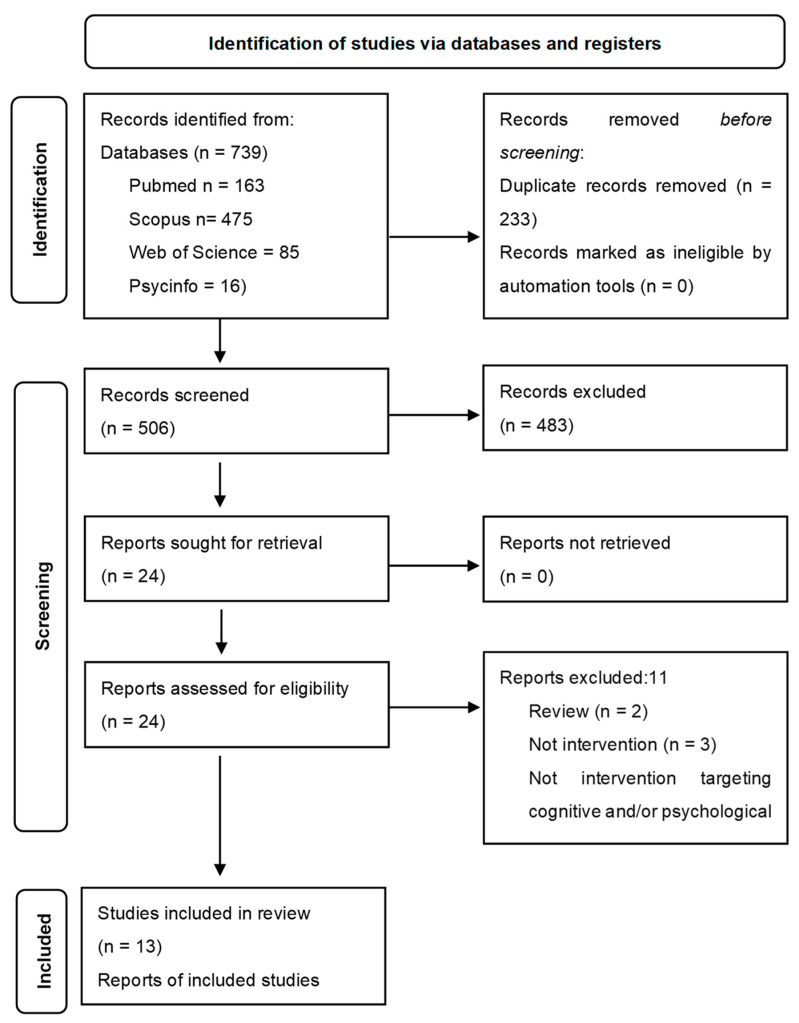
Preferred Reporting Items for Systematic Reviews and Meta-Analyses flowchart of the systematic review.

Our search retrieved 739 articles in total. After removing 233 duplicates, 506 relevant articles were screened based on title and abstract. Of those 506 articles, 483 were excluded, and 24 potentially relevant articles were screened in full text. Of these papers, 11 were excluded based on the inclusion/exclusion criteria. Finally, 13 articles were eligible for inclusion in this review (Figure 1).

### 3.2. Evaluation of the Quality of the Studies

Regarding the evaluation of the quality of the articles included in this systematic review (Table 1), the percentage of compliance with the JBI criteria ranged from 7.69% to 92.31%, depending on the study [53]. There were no specific criteria met by all 13 studies. Nine of the 13 studies analyzed used true randomization for the assignment of participants to treatment groups [28,30,45,54,55,56,57,58,59], whereas the remaining 4 did not meet this criterion [22,24,31,32].

In four of the studies, allocation to treatment groups was concealed [28,45,58,59], with two of them evaluated as unclear in this category [54,55], whereas the remaining seven did not meet this criterion [22,24,30,31,32,56,57].

Regarding the treatment groups, in nine of the studies, these were similar at the baseline [28,30,45,54,55,56,57,58,59], whereas in the remaining four articles, this criterion was not met [22,24,31,32].

In four of the studies, participants were blind to treatment assignments [45,55,56,58], whereas in eight of them, this criterion was not met [22,24,28,31,32,54,57,59], and in one of them this criterion was unclear [30]. Furthermore, in 2 of the studies, those delivering treatment were blind to treatment assignment [28,58]. The remaining 11 studies did not meet this criterion [22,24,30,31,32,45,54,55,56,57,59].

Outcome assessors were blind to treatment assignment in 2 of the studies [28,45]. The remaining 11 studies did not meet this criterion [22,24,30,31,32,54,55,56,57,58,59].

Regarding the treatment groups being treated identically other than the intervention of interest, this was found in nine of the studies [24,28,45,54,55,56,57,58,59], whereas the remaining four articles did not meet this criterion [22,24,31,32].

In only 1 study [32], neither follow up was complete, nor were differences between groups in terms of their follow up adequately described and analyzed. In the 12 remaining studies, this criterion was met [22,24,28,30,31,45,54,55,56,57,58,59].

In 10 studies, participants were analyzed in the groups in which they were randomized [28,30,32,45,54,55,56,57,58,59], whereas the remaining 3 studies failed to meet this criterion [22,24,31].

In nine studies, outcomes were measured in the same way for the different treatment groups [28,30,45,54,55,56,57,58,59]. The remaining four studies failed to do so [22,24,31,32].

We consider that outcomes were measured in a reliable way in eight of the studies [22,24,28,54,55,56,57,58], whereas this criterion was not met in two other studies [30,32], and it is unclear in the other three [31,45,59].

Appropriate statistical analysis was used in 12 of the 13 studies [22,24,28,30,32,45,54,55,56,57,58,59], while this matter is unclear in 1 study [31].

In nine of the studies, the trial design was appropriate, and any deviations from the standard RCT design were accounted for in the conduct and analysis of the trial [28,30,32,45,55,56,57,58,59], whereas in the remaining four studies, this criterion was not met [22,24,31,54].

### 3.3. Sample Characteristics

Participants from all the studies were diagnosed with CKD, and most were undergoing hemodialysis treatment. The sample size at baseline ranged from 8 to 156 individuals, with a total of 824 participants across the studies. In 12 out of the 13 papers (92.3% of the studies), authors provided information about the sex of the participants, and from a total of 770 people, 350 were male and 420 female. Regarding age, two studies (15.3% of the studies) did not specify the mean age of participants; out of the remaining studies (84.7%), the mean age of participants was 56.4 years (see Table 2). Recruitment mostly occurred via medical centers or hospitals.

### 3.4. Description of Electronic Health Interventions

**Figure 2:**
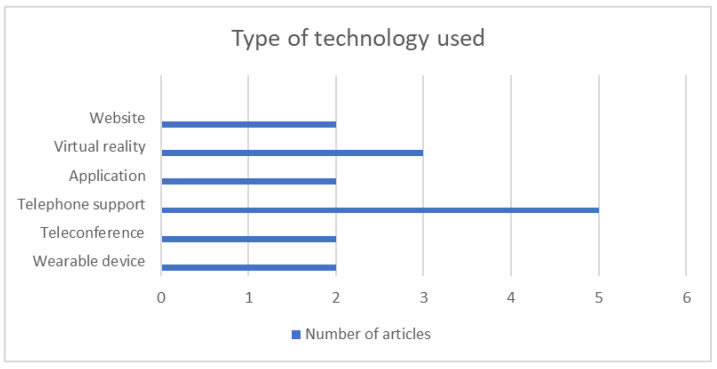
Type of technology used in selected articles.

Major types of eHealth and key components of the intervention are summarized in Table 3. While a minority of studies included multiple components (multiple eHealth type) to monitor or measure patients’ outcomes, 4 of a total of 13 reviewed articles (30.7% of the studies), most of the studies (61.54%) only used one type of technology in the intervention process. The most used intervention component was telephone support, reviewed in 5 of the 13 articles (38.4% of the studies), followed by VR, which was evaluated in 3 of a total of 13 articles (23% of the studies) (Figure 2).

### 3.5. Study Characteristics

**Figure 3:**
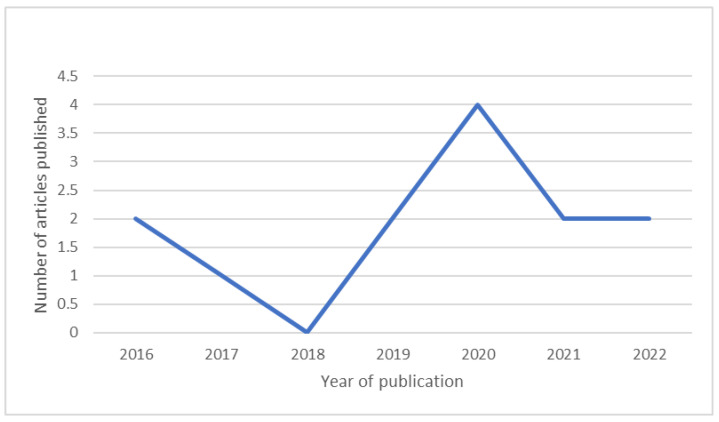
Number of selected articles published between 2016 and 2022.

As mentioned previously, we searched for articles published between 2012 and 2022. However, all 13 studies included in this review were published between 2016 and 2022, with 8 of them (61.5% of the studies) being conducted between 2020 and 2022 (Figure 3).

A total of four articles (30.7% of the studies) were conducted in the United States of America, followed by three (23% of the studies) conducted in Australia and two (15.3% of the studies) in Taiwan. In addition, one study (7.6% of the studies) was conducted in Bangladesh, one (7.6% of the studies) in Brazil, one (7.6% of the studies) in Canada, and one (7.6% of the studies) in Iran. The research designs varied between the studies; most used a randomized controlled trial design. The duration of the interventions ranged from 4 weeks to 6 months. Moreover, many studies included a control group, concretely nine (69.2% of the studies), and a total of six studies (46.1% of the studies) performed follow-up measurements. All the studies focused on the usability, acceptability, or feasibility of eHealth interventions aimed at psychological well-being symptoms, with no studies aiming at cognitive symptoms. The most common psychological symptoms managed were quality of life (eight articles or 61.5% of the studies), depression (six articles or 46.1% of the studies), and self-efficacy (four articles or 30.7% of the studies). Psychological symptoms were measured throughout the studies using a total of 22 different scales, with CES-D [60,61] being the one most used (in four articles or 30.7% of the studies). The main results obtained in the manuscripts reviewed are shown in Table 4.

## 4. Discussion

### 4.1. Main Findings

Relevant data from 13 articles were included, and the evidence regarding the implementation and effectiveness of technology-based interventions for CKD patients, addressing cognitive and psychological symptoms, were reviewed. The year with the largest number of articles published (3) was 2020, and the country with the largest number of articles published (4) was the United States. Most of the articles (10) were purely experimental, two were quasi-experimental, and one of the articles used mixed methods. In all the articles, interventions targeting psychological symptoms were developed using different technological tools. However, we were not able to find any interventions for cognitive symptoms in patients with CKD.

Regarding the sample, the number of participants ranged from 8 [24] to 156 [45]. All the selected articles, apart from one [56], informed about the sex of the sample. In this line, the total number of men participating in the remaining studies was 350, while the number of women was 420. The mean age of the participants ranged from 46.45 to 64.5 years—this data was informed by all the studies apart from two [31,56].

The current review suggests that technology-based interventions offer feelings of safety, fun, and satisfaction, as reported by participants [24,30,32]. Apart from being perceived as safe, these interventions also have the potential to improve CKD patients’ health outcomes regarding their psychological well-being. For instance, psychological distress, anxiety, and depression have been addressed by 7 of the 13 studies included in the present review. Maynard et al. [28], Zhou et al. [30], and Chou et al. [54] used VR exergames in order to address depressive or distress symptoms. It is important to note that whereas the first one showed that virtually supervised intradialytic exergame is as efficient as a nurse-supervised intradialytic exercise in significantly reducing depression symptoms [28], the second research study found that the intervention minimized depressive symptoms, but without showing a significant reduction [30]. According to the authors, this may be due to the duration of the exercise protocol (30–60 min). Nonetheless, comparing the protocol of both studies, we can see that while the study performed by Zhou et al. [30] consisted of 30 min sessions 3 times per week for 4 weeks, the one performed by [28] was much longer, with 30–60-min sessions, 3 times per week for 12 weeks. For this reason, we discard the length of the exercise protocol as a possible reason for these differences in results.

On the other hand, Chou et al. [54] reported a decrease in levels of fatigue as expressed by symptoms of distress, together with a loss of control in mood, reduced vigor and motivation, and mental ability. Other authors addressed depressive symptoms through internet-delivered cognitive-behavioral therapy (CBT). For instance, Jakubowski et al. [24] did not find a significant improvement in depression with his 8-session 8-week intervention. Other authors [22] did find a significant improvement in depression, as well as in anxiety and general distress, with his 5-lesson 8-week intervention. According to these last authors, they used “an existing and efficacious internet-delivered CBT course, called the Wellbeing course, for anxiety and depression” [22]. Mindfulness was also applied as a tool to potentially reduce anxiety and depression symptoms through teleconference workshops. However, the results did not confirm a significant reduction in these symptoms. Despite these results, a large number of participants continued practicing mindfulness and reported that it could be helpful for stress management [55]. Lastly, Dingwall et al. [45] found that talking about their well-being and receiving information relevant to kidney health through culturally adapted applications significantly reduced general distress and depression symptoms in hemodialysis patients, even though the intervention was brief, consisting of two sessions and a phone-call (reminding patients of their goals and addressing barriers to goal attainment).

Health-related quality of life is the variable most widely evaluated, addressed by 8/13 studies included in the review. This variable has been addressed using different means, including telephone support [45,58,59], VR exergames [28], use of applications [45], internet-delivered CBT [22,24], internet-delivered Mindfulness workshops [55], or use of wearables combined with health management platforms and social media [57]. Results indicate that, although they differed in the length of the intervention and the tools used, most interventions succeeded in improving the quality of life of patients. Nonetheless, two studies failed to do so. For example, Sarker et al. [59] found that quality of life changed more favorably in the intervention group, although the difference with the control group did not reach statistical significance. This may be due to the actual length of the intervention, which consisted of 10 min sessions every 2 weeks for 6 months. Dingwall et al. [45] also discussed the importance of the test used to measure quality of life (European Quality of Life -EQ-5D-). However, the utility of this measure is still unclear regarding its ability to tap into the different domains of CKD patients’ quality of life.

Other symptoms addressed were related to perceived self-efficacy and/or self-management and were present in four studies analyzed for the present review. Using a self-management intervention with access to a website with educational content regarding CKD, perceived self-efficacy did not significantly improve for participants. However, their confidence in gathering information about the disease improved [31]. Another investigation [58] was developed in the framework of the social cognitive theory (SCT), which states that human behavior can be modified by improving the person’s self-efficacy—the belief that they can perform that behavior in a successful way [62]. However, their results showed that a large effect size was only observable for improved self-management and self-efficacy scores, which can be due to the fact that self-management is not just an individual issue. Instead, self-management should be considered within families and in the context of social support. The people closer to patients could potentially be actively involved in helping them create a good environment where they can perform everyday activities to manage CKD. Therefore, according to these authors, this fact should be recognized by including family members in self-management education sessions. In this line, further research about the role of family members or caregivers in supporting CKD self-management is needed. Despite these indications, Jahromi et al. [56] and Li et al. [57] conducted studies that significantly improved self-efficacy and self-management perceptions related to decision-making and/or problem-solving in patients with CKD.

Cognitive symptoms are present in 20% to 60% of CKD patients and are linked to a higher risk of developing dementia [63,64]. Moreover, the importance of the development of different strategies to improve cognitive function in these patients has been highlighted by various studies [6,8,39,65]. This being said, in this review, we did not find any studies aimed at exploring the effectiveness of technology-based interventions in improving cognitive symptoms in CKD patients.

### 4.2. Technology-Based Interventions

In the articles included in this systematic review, a great variety of technology-based intervention components were used (e.g., applications, websites, VR, and telephone support), with most of them proving to be effective in improving the psychological well-being of CKD patients. In reviewing the results of the studies, it has been seen that when some interventions tried to increase self-efficacy and knowledge in these patients, the use of certain components such as telephone calls or websites, did not reach statistical significance after the intervention. However, we consider that they could still be promising tools. Regarding VR, the level of immersion can range from virtual to augmented and mixed reality, depending on the equipment and technology used. Thus, based on the immersion level, VR can be fully immersive, semi-immersive, and non-immersive [35,66,67]. In this review, two studies that applied non-immersive VR significantly reduced fatigue and depression [28,30]. Another study that used immersive VR to conduct a mindfulness/meditation program showed a significant decrease in symptoms after exposure [32]. Other components such as applications, teleconferences, or telephone calls proved to be useful in reducing distress, depression, and anxiety, as well as improving health-related quality of life.

On the one hand, the diverseness of technologies seen in this review allows an approximation towards the identification of those types of technologies most frequently used, as well as towards the symptoms targeted. On the other hand, the use of so many different technologies in so few studies makes it difficult to draw definitive conclusions with regard to their efficiency. However, in a systematic review of the use of VR in mental health in older adults, it has been indicated that cognitive training is one of the most supported VR approaches in the literature. Furthermore, it has been anticipated that VR will be used as a platform for delivering interventions and therapies such as mindfulness, exposure therapy, and behavioral activation [68]. Therefore, it would be interesting to further explore VR as a tool in older adults’ mental health, especially when there is a need for extensive research on the application of VR technology for older adults with mood alterations, anxiety responses, and other psychiatric diagnoses.

The age of the sample, as well as their familiarity with new technologies, are two other factors to take into consideration when investigating the effectiveness of technology-based interventions in adults with CKD. In a meta-analysis performed by Hauk et al. [69], it was confirmed that age was negatively related to technology acceptance of various technologies. In this line, the mean age of the participants from the studies selected for the present review ranged from 46.45 to 64.5 years, which could have possibly interfered with the effectiveness of the technology tools.

### 4.3. Evaluation of Study Quality

After evaluating the study quality of the articles included in this review applying JBI criteria (The Critical Appraisal Checklist for Randomized Controlled Trials-RCTs-), it has been seen that the percentage of compliance with the JBI criteria ranged from 7.69% to 92.31%, with a mean percentage of 58.58%. There were no specific criteria met by all the studies. However, nine criteria were met in nine or more studies (e.g., the treatment groups were similar at the baseline, outcome assessors were blind to treatment assignments, and participants were analyzed in the groups in which they were randomized). This would reflect that a high percentage of the 13 studies reviewed displayed good quality, although some had limitations in some of the evaluated sections.

Of note, four studies with a percentage of compliance of 23.08% or less were included. One might argue that such studies should be excluded based only on the percentage of compliance. However, high-level evidence on the effectiveness of technology-based interventions addressing psychological and/or cognitive symptoms in CKD patients, for instance, generated by large RCTs, is very limited. Hence, studies with less percentage of compliance are included since, in this stage, we believe that all experimental evidence should be added and taken into account in order to deepen our understanding of the usability and effectiveness of technology-based interventions for CKD patients. In general, recent studies show higher levels of quality, and it is expected that better evidence of the use of technologies applied to chronic and neurodegenerative diseases can be obtained in the coming years [70].

### 4.4. Strengths and Limitations

As far as we know, this is the first systematic review to explore the effects of technology-based interventions addressing cognitive and psychological symptoms in patients with CKD. Our study has several strengths: (1) PRISMA guidelines were followed; (2) in accordance with the PRISMA guidelines, a rigorous selection of the studies reviewed was carried out based on inclusion and exclusion criteria; (3) a complete search strategy was done in four electronic databases specialized in health sciences: MEDLINE/PubMed, Scopus, Web of Science, and ProQuest; and (4) a comprehensive analysis of the 13 final articles on the intervention components, outcome measures and determinants from the studies were performed. Our methodology also has some limitations that must be considered: (1) by including only articles in the English language and papers published from 2012 onwards, we may have left out relevant articles in other languages or earlier relevant literature. However, given that electronic-health and new technologies interventions are relatively new, we believe that the research timeframe used (between 2012 and 2022) captures the most relevant studies; and (2) our search period ended in December 2022, and we are aware that an update will be required in futures reviews, as more studies on the effects of technology-based interventions will be published.

### 4.5. Conclusions

The present systematic review shows that the technology-based interventions identified in the search performed in different databases suggest that this non-pharmacological approach may be useful in the management of psychological well-being symptoms of patients with CKD, including those undergoing hemodialysis. The symptoms most successfully managed by technological applications are depression, anxiety, health-related quality of life, fatigue, self-efficacy, and self-management. Regarding cognitive symptoms, we did not find any studies using technology-based interventions aimed at improving cognitive functioning in patients with CKD.

The use of technological strategies in interventions has been shown to increase motivation and adherence to treatment in CKD patients. The results obtained in this systematic review may, on the one hand, promote the development of new interventions that allow patients to carry out parallel work during hemodialysis treatment. On the other hand, these results may also highlight new ways of improving health-related quality of life from home, using entertaining, efficient, and low-cost strategies.

The results obtained in this review show that studies regarding the effects of technology-based interventions on the cognition and psychological well-being of people with CKD are scarce. Moreover, after being informed about the cognitive impairment in CKD patients and observing the lack of technology-based interventions in treating this impairment, the importance of maintaining cognitive functioning and preventing its decline is becoming increasingly apparent. Therefore, in order to adequately assess the technology-based health interventions effect, future lines of research should consider designing non-pharmacological treatments for the improvement of cognitive and psychological symptoms in this type of patient.

## Figures and Tables

**Table 1:** Risk of bias of RCTs.

Authors	Q1	Q2	Q3	Q4	Q5	Q6	Q7	Q8	Q9	Q10	Q11	Q12	Q13	% of Compliance
Chan et al. (2016) [22]	N	N	N	N	N	N	N	Y	N	N	Y	Y	N	23.08%
Jahromi et al. (2016) [56]	Y	N	Y	Y	N	N	Y	Y	Y	Y	Y	Y	Y	76.92%
Gross et al. (2017) [55]	Y	U	Y	Y	N	N	Y	Y	Y	Y	Y	Y	Y	76.92%
Maynard et al. (2019) [28]	Y	Y	Y	N	Y	Y	Y	Y	Y	Y	Y	Y	Y	92.31%
Nguyen et al. (2019) [58]	Y	Y	Y	Y	Y	N	Y	Y	Y	Y	Y	Y	Y	92.31%
Chou et al. (2020) [54]	Y	U	Y	N	N	N	Y	Y	Y	Y	Y	Y	N	61.54%
Jakubowski et al. (2020) [24]	N	N	N	N	N	N	N	Y	N	N	Y	Y	N	23.08%
Li et al. (2020) [57]	Y	N	Y	N	N	N	Y	Y	Y	Y	Y	Y	Y	69.23%
Zhou et al. (2020) [30]	Y	N	Y	U	N	N	Y	Y	Y	Y	N	Y	Y	61.54%
Dingwall et al. (2021) [45]	Y	Y	Y	Y	N	Y	Y	Y	Y	Y	U	Y	Y	84.62%
Hernandez et al. (2021) [32]	N	N	N	N	N	N	N	N	Y	N	N	Y	Y	23.08%
Donald et al. (2022) [31]	N	N	N	N	N	N	N	Y	N	N	U	U	N	7.69%
Sarker et al. (2022) [59]	Y	Y	Y	N	N	N	Y	Y	Y	Y	U	Y	Y	69.23%

Abbreviations: N: No; U: Unclear; Y: Yes; Q1: Was true randomization used for assignment of participants to treatment groups?; Q2: Was allocation to treatment groups concealed?; Q3: Were treatment groups similar at the baseline?; Q4: Were participants blind to treatment assignment?; Q5: Were those delivering treatment blind to treatment assignment?; Q6: Were outcome assessors blind to treatment assignment?; Q7: Were treatment groups treated identically other than the intervention of interest?; Q8: Was follow-up complete, and, if not, were differences between groups in terms of their follow-up adequately described and analyzed?; Q9: Were participants analyzed in the groups to which they were randomized?; Q10: Were outcomes measured in the same way for treatment groups?; Q11: Were outcomes measured in a reliable way?; Q12: Was appropriate statistical analysis used?; Q13: Was the trial design appropriate, and any deviations from the standard RCT design (individual randomization, parallel groups) accounted for in the conduct and analysis of the trial?

**Table 2:** Sociodemographic characteristics of participants per study (number of participants, sex, mean age, and CKD status).

Study	Number of Participants	Men	Women	Mean Age	Status
Chan et al. (2016) [22]	*n* = 22	16 (72.7%)	6 (27.3%)	59 years	CKD HD
Jahromi et al. (2016) [56]	*n* = 54	Not specified	Not specified	Not specified	CKD HD
Gross et al. (2017) [55]	*n* = 55	24 (43.6%)	31 (56.4%)	54 ± 12 years	CKD
Maynard et al. (2019) [28]	*n* = 40	22 (55%)	18 (45%)	46.45 ± 13.5 years	CKD HD
Nguyen et al. (2019) [58]	*n* = 135	68 (50.4%)	67 (49.6%)	48.85 ± 13.8 years	CKD stages 3–5 (no dialysis)
Chou et al. (2020) [54]	*n* = 64	32 (50%)	32 (50%)	55 ± 9.4 years	CKD HD
Jakubowski et al. (2020) [24]	*n* = 8	4 (50%)	4 (50%)	59 years	CKD HD
Li et al. (2020) [57]	*n* = 49	36 (73.5%)	13 (26.5%)	64.5 ± 8.7 years	CKD stages 1–4
Zhou et al. (2020) [30]	*n* = 73	33 (45.2%)	40 (54.8%)	64.5 ± 8.7 years	CKD HD
Dingwall et al. (2021) [45]	*n* = 156	44 (28.2%)	112 (71.8%)	55 ± 9.4 years	CKD HD
Hernandez et al. (2021) [32]	*n* = 20	16 (80%)	4 (20%)	55.3 ± 13.1 years	CKD HD
Donald et al. (2022) [31]	*n* = 22	12 (54.6%)	10 (45.4%)	Not specified	CKD (no dialysis)
Sarker et al. (2022) [59]	*n* = 126	43 (34.1%)	83 (65.9%)	55.3 ± 13.1 years	CKD

Abbreviations: CKD (Chronic Kidney Disease); HD (Hemodialysis).

**Table 3:** Main characteristics of the included papers: authors (year), country, design, length of study, psychological and/or cognitive measures, other measures, type of technology used, and psychological and/or cognitive symptoms managed.

Authors	Country	Design	Treatment Length and Description	Psychological Well-Being/Cognitive Measures	Other Measures	Type of Technology Used	Psychological/ Cognitive Symptoms Managed
Chan et al. (2016) [22]	Australia	Experimental, single-group uncontrolled open trial design with symptom assessment at pre-treatment, immediately post-treatment, and follow-up assessment. *n* = 22	5 lessons with a total duration of 8 weeks (cognitive and behavioral skills for psychological distress management).	PHQ-9 GAD-7 K10 SDS SF12v2 MINI	KDLS BKDS	iCBT using telephone calls and emails.	General psychological distress, depression, anxiety, quality of life, and kidney disease-related loss.
Jahromi et al. (2016) [56]	Iran	Single-blind randomized clinical trial. Self-care training group (CG): *n* = 30 Self-care training with telephone follow-up group (IG): *n* = 30	CG: information not available. IG: 24 phone calls with a duration of 20 min each (in 8 weeks) and 5 instructional sessions and instruction booklet.	SUPPH		Telephone assistance.	Self-efficacy (positive attitudes, stress, and decision-making).
Gross et al. (2017) [55]	United States of America	Experimental, randomized, active-controlled, open-label trial with a follow-up assessment. tSupport (active CG): *n* = 28 tMBSR (IG): *n* = 27	CG: two 1.5 h workshops and six 1 h weekly teleconferences. IG: 1 session per week for a total of 8 weeks. In-person 3 h workshops in w1 and w8 and 1.5-h group teleconferences in w2 to w7.	STAI SF-12v2 (PCS, MCS, and pain interference item) CES-D PSQI PROMIS-Fatigue Short Form		Workshop in teleconference format.	Anxiety, depression, fatigue, and health-related quality of life.
Maynard et al. (2019) [28]	Brazil	Experimental, randomized controlled trial. CG: *n* = 20 IG: *n* = 20	CG: maintained only HD. IG: performed exergames 30–60 min-sessions, 3 times per week for 12 weeks.	KDQoL-SF CES-D	Blood pressure, respiratory rate, pulse, and oxygen saturation. Walking speed. Timed up and go. DASI	VR games.	Quality of life and depressive symptoms.
Nguyen et al. (2019) [58]	Australia	Experimental, single-blind pragmatic randomized controlled trial with 1:1 allocation into two parallel groups with repeated measures and follow-up assessment. CG: *n* = 67 IG: *n* = 68	CG: usual CKD care. IG: 12-week self-management intervention (CKD booklet, a handout, 1 face-to-face session, and 2 follow-up sessions). Follow-up assessment at w4 and w12.	CKD-SM KiKS SECD SF-36v2		tSupport.	Self-management behavior, self-efficacy, and health-related quality of life.
Chou et al. (2020) [54]	Taiwan	Quasi-experimental study design to analyze pre- and post-test measures. CG: *n* = 32 IG: *n* = 32	CG: usual routine care (advice on physical activities). IG: 30 min VR sessions, 3 times per week for 4 weeks.	NFSHD		VR exercise program using Nintendo Wii Fit.	Fatigue (reduction in vigor and motivation, mental ability, distress, loss of control in mood).
Jakubowski et al. (2020) [24]	United States of America	Experimental, single-center pilot feasibility study with a follow-up assessment. *n* = 10	One 45–60 min-session per week for 8 weeks.	FACIT-F CES-D SF-36 (PCS and MCS summary scores)		Online video-conferencing platform (Vidyo).	Fatigue, depression, and quality of life.
Li et al. (2020) [57]	Taiwan	Experimental, two-arm randomized controlled trial with a pretest-posttest design. CG: *n* = 30 IG: *n* = 30	Wearables were given to all the participants, and they maintained dietary diaries using a smartphone app. IG: additionally, they had 90 days of diet, exercise, and self-management education and interacted with each other through a social media group.	KDQoL SF-36 The self-efficacy questionnaire The self-management questionnaire		Wearable devices (wristband). A health management platform (LINE) application	Self-efficacy (problem-solving, partnership), self-management (partnership, compliance, self-care, and problem-solving), quality of life (cognitive function, sexual function, sleep, quality of social interaction).
Zhou et al. (2020) [30]	United States of America	Experimental, randomized controlled trial. SG: *n* = 36 EG: *n* = 37	SG: 30 min weightless foot rotation intradialytic exercise program without technology. EG: 30 min intradialytic exergame sessions, 3 times per week for 4 weeks.	CES-D	TAM Revised	Intradialytic exergame (virtually supervised) and working memory (dual task). Wearable sensors for foot rotation and laptop.	Depressive symptoms
Dingwall et al. (2021) [45]	Australia	Experimental, three-arm, waitlist, single-blind randomized controlled trial with 2:2:1 allocation ratio and follow-up assessment. Treatment as usual/DSS treatment after 3 months (TAU/DSS): *n* = 33 Contact control/DSS treatment (HepB/DSS): *n* = 61 Immediate treatment (ISS): *n* = 62	TAU/DSS: Questionnaires at baseline + follow-up assessment using SS app. Hep B/DSS: 20-min contact with researcher at baseline + 20-min session after 2–4 weeks + follow-up assessment using SS app. ISS: 20 min interview at baseline + 20 min session within 2–4 weeks + follow-up assessment.	K10 Adapted PHQ-9 EQ-5D		ISS App Hep B App Text message or phone call	Psychological distress, depressive symptoms, and quality of life (self-care, anxiety, and depression).
Hernandez et al. (2021) [32]	United States of America	Experimental, single-arm proof-of-concept trial. *n* = 20	25 min program repeated on two separate occasions during consecutive HD treatment sessions (1 month).		SSQ IPQ SUS	Joviality fully immersive VR mindfulness/meditation program.	Discomfort, safety, and acceptability.
Donald et al. (2022) [31]	Canada	Explanatory sequential mixed-methods study with a follow-up assessment. *n* = 33	8 weeks access to MKMH website and 30 min. telephone interviews within 1 month.	CDSES	eHEALS TAM Google Analytics	MKMH website Telephoneinterviews	Self-efficacy.
Sarker et al. (2022) [59]	Bangladesh	Experimental, parallel-group randomized controlled trial with a follow-up assessment. CG: *n* = 63 IG: *n* = 63	CG: standard treatment. IG: health education for 6 months, with phone calls once every 2 weeks.	EQ-5D	KiKN	Health education over a mobile phone call using mHealth technology.	Quality of life and motivation for a healthy lifestyle.

Abbreviations: BKDS (Burden Kidney Disease Scale); CDSES (Chronic Disease Self-Efficacy Scale); CES-D (Center for Epidemiologic Studies Depression Scale); CG (Control Group); CKD (Chronic Kidney Disease); CKD-SM (CKD Self-Management modified); DASI (Duke Activity Status Index); DSS (Delayed Stay Strong); EG (Exercise Group); eHEALS (eHealth Literacy Scale); EQ-5D (European Quality of Life); FACIT-F (Functional Assessment of Chronic Illness Therapy Fatigue); GAD-7 (Generalized Anxiety Disorder 7-ItemScale); Hep B (The Hep B Story); iCBT (Internet-delivered Cognitive Behavioral Therapy); IG (Intervention Group); IPQ (IGroup Presence Questionnaire); ISS (Immediate Stay Strong); K10 (Kessler Distress Scale); KDLS (Kidney Disease Loss Scale); KDQoL (Kidney Disease Quality of Life); KDQoL-SF (Kidney Disease and Quality-of-Life Short-Form); KiKN (Kidney Knowledge Questionnaire); KiKS (Kidney Disease Knowledge Survey); MCS (Mental Component Summaries); Min (minutes); MINI (Mini International Neuropsychiatric Interview); MKMH (My Kidneys My Health); NFSHD (Novel Fatigue Scale for Hemodialysis); PCS (Physical Component Summaries); PHQ-9 (Patient Health Questionnaire-9 Item); PSQI (The Pittsburgh Sleep Quality Index); SDS (Sheehan Disability Scale); SECD (Self-Efficacy for Managing Chronic Disease); SF-12v2 (Short Form 12); SF-36 (Short Form 36 Health Survey); SF-36v2 (Short Form 36); SG (Supervised Exercise Group); SS (Stay Strong); SSQ (Simulator Sickness Questionnaire); STAI (State Trait Anxiety Inventory); SUPPH (Strategies Used by Patients to Promote Health); SUS (System Usability Scale); TAM (Technology Acceptance Model); TAU (Treatment as Usual); tMBSR (Telephone-adapted Mindfulness-based Stress Reduction); tSupport (Telephone Support); VR (Virtual Reality); W (Week).

**Table 4:** Results and conclusions of included papers.

Study	Results	Conclusions
Chan et al. (2016) [22]	Clinically significant improvements in depression, anxiety, and general distress were maintained or further improved by follow-up. Improvements also observed in quality of life and kidney disease-related loss. No improvements in disability and kidney disease burden.	iCBT is an innovative way of increasing access to effective psychological treatment for CKD patients.
Jahromi et al. (2016) [56]	Improved self-efficacy for IG, overall self-efficacy scores, stress reduction, and decision-making.	A combination of self-care training and telephone follow-up improves self-efficacy in HD patients.
Gross et al. (2017) [55]	No significant reductions in anxiety for either group. tMBSR significantly improved mental HRQoL at follow-up for IG.	tMBSR was effective for improving mental HRQoL.A large percentage of tMBSR participants practiced mindfulness and reported it was helpful for stress management.
Maynard et al. (2019) [28]	IG improved significantly in relation to the CG in domains of HRQoL, such as effects of kidney disease, physical functioning, and physical role. No significant differences in depressive symptoms.	Physical training combined with VR improved functional capacity and some HRQoL domains.
Nguyen et al. (2019) [58]	Scores for HRQoL components of physical and mental health significantly improved in the IG at w16. Large effect sizes for improved self-management and self-efficacy were detected at w16.	A self-management intervention improved self-management behavior, self-efficacy, and HRQoL for patients with CKD.
Chou et al. (2020) [54]	Significantly lower levels of overall fatigue, reduced vigor and motivation, lower distress, and loss of control in mood for IG.	Overall fatigue of patients in both groups declined at the post-test, although this was greater for IG.
Jakubowski et al. (2020) [24]	Statistically significant improvement on the SF-36 physical component score (a measure of HRQoL) at follow-up. No statistically significant improvement in depression at follow-up.	The technology-assisted CBT intervention was feasible, well-accepted, and required minimal additional resources.
Li et al. (2020) [57]	Self-efficacy, self-management, and KDQoL scores were significantly higher in the IG at the post-test.	The use of wearable devices, a health management platform, and social media strengthened self-efficacy and self-management and improved quality of life.
Zhou et al. (2020) [30]	Significant reduction in depression scores for both groups. No between-group difference for the observed effect. Positive intradialytic exercise experience expressed by EG, including fun, safety, and helpfulness of sensor feedback.	The virtually supervised low-intensity intradialytic exergame is feasible during HD treatment.
Dingwall et al. (2021) [45]	Significant decreases in K10 and PHQ-9 scores at 3 and 6 months for the HepB/DSS group were seen. For moderate to severe symptoms of distress or depression, significant decreases in K10 and PHQ-9 scores for ISS and HepB/DSS were seen. No significant differences for EQ-5D.	Talking to others about well-being and providing information related to kidney health using culturally adapted apps improves the well-being of people undergoing dialysis.
Hernandez et al. (2021) [32]	Significant decreases in treatment and/or motion-related symptoms after first VR exposure.	VR program decreased symptom severity without adverse effects. Perception involved a relaxing/calming environment, effective tools for active distraction, pleasant scenery, and valuable mindfulness education.
Donald et al. (2022) [31]	High acceptance and increase in CKD information.	MKMH website provides accessible content and tools that may improve self-efficacy and support in CKD self-management.
Sarker et al. (2022) [59]	No significant differences after the intervention; however, QoL, on average, changed more favorably in the IG than in the CG.	A campaign and mobile health as an education strategy showed promise for enhancing CKD knowledge.

Abbreviations: CBT (Cognitive Behavioral Therapy); CES-D (Center for Epidemiologic Studies Depression Scale); CG (Control Group); CKD (Chronic Kidney Disease); DSS (Delayed Stay Strong); EG (Exercise Group); EQ-5D (European Quality of Life); HD (Hemodialysis); Hep B (The Hep B Story); HRQoL (Health-Related Quality of Life); iCBT (Internet-delivered Cognitive Behavioral Therapy); IG (Intervention Group); K10 (Kessler Distress Scale); KDLS (Kidney Disease Loss Scale); KDQoL (Kidney Disease Quality of Life); MKMH (My Kidneys My Health); PHQ-9 (Patient Health Questionnaire-9 Item); SF-36 (Short Form 36 Health Survey); tMBSR (Telephone-adapted Mindfulness-based Stress Reduction); VR (Virtual Reality); W (Week).

## Data Availability

Not applicable.
